# TRIMming down Mycobacterium tuberculosis replication: TRIM32 is required for bacterial ubiquitination and autophagy induction in macrophages

**DOI:** 10.1080/27694127.2023.2278120

**Published:** 2023-11-05

**Authors:** Alessandra Romagnoli, Martina Di Rienzo, Mauro Piacentini, Gian Maria Fimia

**Affiliations:** a Department of Epidemiology, Preclinical Research and Advanced Diagnostics, National Institute for Infectious Diseases IRCCS ‘L. Spallanzani’, Rome, Italy; b Department of Biology, University of Rome ‘Tor Vergata’, Rome, Italy; c Department of Molecular Medicine, University of Rome “La Sapienza”, Rome, Italy

**Keywords:** Xenophagy, TRIM ubiquitin ligases, *Mycobacterium tuberculosis*, Ubiquitin

## Abstract

*Mycobacterium tuberculosis* (Mtb) promotes its intracellular persistence by subverting defense mechanisms, such as autophagy. Remarkably, enhancing autophagy is sufficient to trigger intracellular Mtb killing and effective immune response, making this process a valid target of host-directed therapies. However, several aspects of autophagy regulation during Mtb infection remain unsolved. Tripartite motif (TRIM) proteins are a large family of ubiquitin ligases primarily involved in innate immunity by regulating inflammation and autophagy. By combining transcriptomic and infectivity screens, we recently identified a set of TRIMs that modulate Mtb replication. In detail, overexpression of TRIM22 and TRIM32 reduces Mtb growth in THP1 macrophages, while that of TRIM36 and TRIM56 promotes Mtb replication. Analysis of the molecular mechanisms underlying inhibition of Mtb replication by TRIM32 showed that its overexpression promote xenophagy, a selective autophagy of pathogens, by increasing Mtb ubiquitination and the recruitment of CALCOCO2/NDP52 (calcium binding and coiled-coil domain 2) and MAP1LC3B (microtubule-associated protein 1 light chain 3B) to intracellular bacteria. Consistently, TRIM32 downregulation reduces the xenophagic response, resulting in increased Mtb replication. Altogether, we characterized a novel role for TRIM32 in the host response to pathogen infections and identify TRIM36 and TRIM56 as possible host factors required for Mtb infection.

**Abbreviations:** CALCOCO2/NDP52, calcium binding and coiled-coil domain 2; AMBRA1, activating molecule In BECN1-regulated autophagy; BECN1, BECLIN-1; MAP1LC3B, microtubule-associated proteins 1 light chain 3B; Mtb, *Mycobacterium tuberculosis*; TRIM proteins, tripartite motif proteins; PRKN/PARKIN, parkin RBR E3 ubiquitin protein ligase; CFU: colony forming units; SMURF1, SMAD specific E3 ubiquitin protein ligase 1; STING, Stimulator of interferon genes protein; TLR, Toll-like receptor; SAR selective autophagy receptor.

Tuberculosis is a global health calamity caused by Mtb infections. Most individuals exposed to Mtb mount an efficient immune response culminating in latent infections without clinical signs of disease, in which bacteria remain dormant within alveolar macrophages surrounded by a variety of immune and non-immune cells, forming a structure named granuloma. The remaining 5- 10% of individuals progress to an active disease in their lifetime, in which Mtb escapes from the granuloma and spreads within the lungs and even to other tissues. The reasons underlying the active disease progression remain poorly understood, highlighting the need of in-depth understanding of the molecular mechanisms responsible for the development of a protective immune response.

Autophagy is mostly relevant in this context, not only because it can directly target Mtb for lysosomal degradation, but also considering the relevance of autophagy in regulating immunity. Indeed, autophagy controls inflammation and antigen processing/presentation, which, in turn, influence adaptive immune responses that, if effective, contain Mtb replication avoiding disease development or, if inefficient, fail to limit Mtb growth resulting in an extensive tissue damage.

TRIM (Tripartite motif) proteins are a family of ubiquitin ligases with more than 80 members, involved in several cellular activities, ranging from signal transduction to transcriptional regulation. Numerous experimental data support a major role of TRIMs in innate immunity, in which they control inflammation and autophagy. For instance, TRIMs directly contribute to xenophagy by detecting pathogens and triggering autophagosome-mediated sequestration in a both ubiquitination-dependent and -independent manner. A role of TRIMs in regulating xenophagy during Mtb infection has recently been emerging. A relevant example is represented by TRIM16, which ubiquitinates Mtb-containing phagosomes and promote their autophagosomal engulfment. In particular, TRIM16 is recruited to phagosomes by GALECTIN-3, which binds to intraluminal glycans that are exposed when the vesicle membrane is damaged by the bacterial effectors released by their secretion systems. However, a comprehensive and systematic analysis of the role of TRIM proteins in Mtb infection has not been done.

To identify TRIM proteins involved in Mtb infection, we recently carried out a transcriptomic analysis combined to cell-based infectivity screens^1^. Specifically, monocyte-derived macrophages were exposed to Mtb strain H37Rv or BCG and transcriptomic changes evaluated by RNAseq. Differential expression analysis identified 17 *TRIM* genes (*TRIM2, 3, 5, 6, 9, 14, 21, 22, 32, 34, 36, 44, 55, 56, 65, 68, 69*) whose levels are altered upon infection. To evaluate the relevance of these TRIMs in Mtb infection, we generated GFP fusion of each of them in lentiviral vectors and used them to transduce THP1 monocytes. Cells were then differentiated into macrophages and infected with Mtb H37Rv-DsRed, and the frequency of intracellular bacteria evaluated by confocal analysis. Gene overexpression was preferred to gene silencing because most of the modulated transcripts of the selected TRIM proteins were downregulated upon Mtb infection. We found that overexpression of TRIM22 and TRIM32 reduces Mtb infection in THP1, while overexpression of TRIM36 and TRIM56 has the opposite effect, favoring Mtb replication. We then focused our investigation on the molecular mechanisms by which TRIM32 modulates Mtb infection. We found that this protein potentiates autophagy flux in infected macrophages, which correlates with an increase of Mtb ubiquitination, recruitment of the selective autophagy receptor (SAR) CALCOCO2 to Mtb and colocalization of Mtb with the autophagosomal marker MAP1LC3B. Notably, we also demonstrated that autophagy activity is required for restricting Mtb infection by TRIM32, by evaluating colony forming units (CFU) in cells silenced for BECN1 (BECLIN-1) or AMBRA1 (Activating molecule In BECN1-regulated autophagy) expression. Consistently, we observed that TRIM32 downregulation leads to increased Mtb infection rate when evaluated by CFU. This is associated to an impaired xenophagic response both in terms of autophagy flux levels and colocalization of Mtb with ubiquitin, CALCOCO2, MAP1LC3B and BECN1, underlying a key role of TRIM32 in signaling the presence of Mtb to the autophagy machinery (Figure 1).

**Figure 1.**
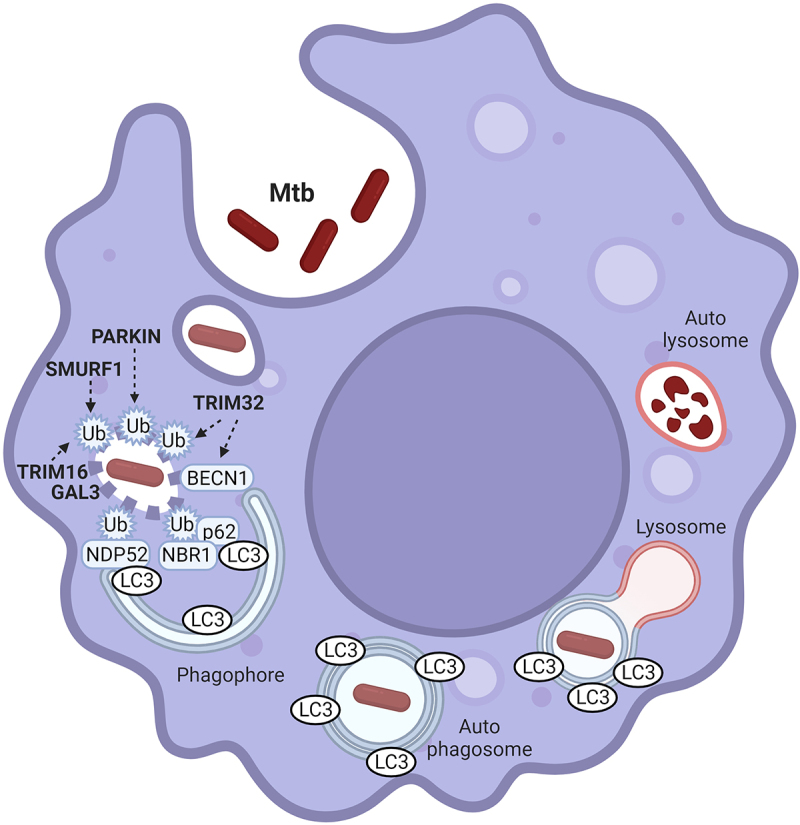
**Molecular mechanisms underlying xenophagy response to Mtb infection**. Intracellular Mtb replication and survival in macrophages is counteracted by xenophagy-mediated lysosomal degradation. Membrane permeabilization by bacterial effectors makes Mtb-containing vesicles accessible to the autophagy machinery. In this context, Mtb has to be first ubiquitinated to then recruit the SARs of the sequestome 1 (SQSTM1)/P62 family. SARs are both involved in recruiting the autophagy machinery for the in situ formation of the autophagosome (not shown in the picture), and in bacterial engulfment by binding to the autophagosome protein MAP1LC3B (LC3). PARKN, SMURF1, TRIM16 and TRIM32 are E3 ligases mediating Mtb ubiquitination. TRIM32 is also necessary to allow BECN1 translocation onto Mtb-containing vesicles. GAL3: GALECTIN 3. Phagophore: nascent autophagosome. The picture was created with BioRender.com.

Previous studies have characterized SMURF1 (SMAD specific E3 ubiquitin protein ligase 1), TRIM16 and PARKN (Parkin RBR E3 ubiquitin protein ligase) as ubiquitin ligases involved in Mtb ubiquitination and xenophagy-mediated degradation, with each of these factors responsible for the ubiquitination of a distinct Mtb population. Although the reason for this redundancy remains to be understood, one possibility is that macrophages employ different ubiquitin ligases upon activation of distinct pathogen sensors. Elucidating the signaling pathways that activate PARKN, SMURF2 and TRIM32 upon Mtb infection will represent an important task to understand how the xenophagic response is concerted in infected macrophages. Interestingly, TRIM32 was reported to act downstream to TLR(toll-like receptor)3, TLR4 and STING (Stimulator of interferon genes protein), which are key sensors able to control Mtb infection. The elucidation of these regulatory events will be of great relevance for elucidating novel aspects of the innate immune response against Mtb infection and for developing host-directed therapies to treat tuberculosis and other microbial diseases.
